# Cognitive Remediation in Middle-Aged or Older Inpatients with Chronic Schizophrenia: A Randomized Controlled Trial in Korea

**DOI:** 10.3389/fpsyg.2017.02364

**Published:** 2018-02-06

**Authors:** Kee-Hong Choi, Jinsook Kang, Sun-Min Kim, Seung-Hwan Lee, Seon-Cheol Park, Won-Hye Lee, Sun Choi, Kiho Park, Tae-Yeon Hwang

**Affiliations:** ^1^Department of Psychology, Korea University, Seoul, South Korea; ^2^Department of Psychiatry, Inje University College of Medicine and Ilsan Paik Hospital, Goyang, South Korea; ^3^Department of Psychiatry, Inje University College of Medicine and Haeundae Paik Hospital, Busan, South Korea; ^4^Department of Clinical Psychology, National Center for Mental Health, Seoul, South Korea; ^5^Department of Clinical Psychology, Yongin Mental Hospital, Yongin, South Korea; ^6^Division of Mental Health Service and Planning, National Center for Mental Health and Yongin WHO Collaborating Center for Psychosocial Rehabilitation and Community Mental Health, Seoul, South Korea

**Keywords:** cognitive remediation, older patients, brain plasticity, schizophrenia, inpatient psychiatric rehabilitation

## Abstract

**Background:** Accumulating evidence indicates that cognitive remediation (CR) is effective for improving various cognitive deficits in adult patients with schizophrenia. Although reports of brain plasticity in older adults and the service needs for chronic patients with schizophrenia are increasing, very few randomized controlled trials of CR have been conducted in middle-aged or older inpatients with chronic schizophrenia. We investigated the efficacy of individualized CR on the cognitive impairments of middle-aged or older inpatients with chronic schizophrenia within the context of comprehensive psychiatric rehabilitation (PR) by comparing the results obtained with PR only and treatment as usual (TAU).

**Method:** Fifty-seven middle-aged and older individuals with chronic schizophrenia and mild to moderate cognitive deficits were enrolled. Thirty-eight who were undergoing PR were randomly assigned to CR + PR (*N* = 19) or PR-only (*N* = 19) groups. Nineteen participants who were undergoing TAU without CR or PR were evaluated pre- and post-treatment.

**Results:** CR was easily provided and well received (drop-out rates = 5.3%) by middle-aged or older psychiatric inpatients. Compared to the PR-Only or TAU patients, patients in the CR + PR group showed greater improvement in executive functioning. Compared to TAU patients, CR + PR and PR-only patients showed greater improvement in logical memory. More patients in the CR + PR group improved clinically significantly in executive functioning and logical memory, compared with the PR-only and TAU patients.

**Conclusions:** These results suggested that CR improved some cognitive deficits in middle-aged or older inpatients with chronic schizophrenia and that it was effective as an adjunctive treatment to the usual PR services provided in inpatient settings.

**Clinical Registration:** KCT0002609

## Introduction

Cognitive deficits have been identified as a key predictor of the functioning of patients with schizophrenia across various phases of the illness from the first episode to chronic illness, even after considering the patients' psychiatric symptoms (Green, 1996; Green and Nuechterlein, 1999; Green et al., 2004; Kurtz et al., 2005; Keefe et al., 2006; Rund et al., 2007). Cognitive functioning has critical therapeutic implications in diverse contexts, including treatment (Smith et al., 1999; Lysaker and Buck, 2007), and vocational rehabilitation (Bell et al., 2007, 2014; McGurk et al., 2015).

Given the therapeutic and functional importance of cognitive deficits in this population (Keefe and Harvey, 2012), cognitive remediation (CR) has received substantial attention as an adjunct treatment option for psychiatric rehabilitation (PR) (Wykes and Spaulding, 2011). In recent decades, randomized controlled trials (RCTs) have demonstrated the efficacy of CR on psychiatric symptoms, cognition, and functioning (Krabbendam and Aleman, 2003; McGurk et al., 2007a; Grynszpan et al., 2011; Wykes et al., 2011).

With the recent increases in life expectancy, the service needs for chronic mid-aged or older adults with chronic schizophrenia are growing (Granholm et al., 2005; Bartels et al., 2014; Schoepf et al., 2014). Cognitive function is responsible for functional competence even after considering key demographic information, such as age and education, and the anticholinergic burden of medications, in older adults with schizophrenia (Tsoutsoulas et al., 2016).

Even though older adults have brain plasticity (Willis et al., 2006; Boron et al., 2007), it is unclear whether CR is effective in middle-aged or older adults with or without schizophrenia (Kontis et al., 2013) or the benefits of CR are also produced by untrained cognitive tasks (Owen et al., 2010).

Even though CR has been reported as more beneficial to younger patients with schizophrenia compared to mid-aged or older patients with schizophrenia (McGurk and Mueser, 2008; Kontis et al., 2013), several studies have indicated that CR still has benefits for in mid-aged or older patients. Wykes and colleagues (Wykes et al., 2007) have reported that, after approximately 30 h of cognitive training, patients who were 40 or older had similar improvements in their memory as those shown by patients younger than 40. Bowie et al. (2014) have reported that older patients (mean age, 45.4) showed improvements in verbal memory and verbal fluency as younger patients (mean age, 28.1) did, while only the younger patients had improvements in processing speed and executive functioning. Most recently, Corbera et al. (2017) conducted a secondary analysis of the results of three different RCTs to investigate the responses of patients with three different age ranges (i.e., <26, between 26 and 39, and over 40) to CR treatment, and they reported that the younger patients benefited more (trend level of significance) compared with the older patient group.

Because promising findings have been reported in non-RCTs, a RCT of the role of CR in the treatment of the cognitive functioning of middle-aged or older patients with schizophrenia is still needed. Sharma et al. (2016) highlighted in their review that controlled studies are needed to better understand whether CR benefits older adults with severe mental illness within the context of broad PR treatments, even though CR appears to have therapeutic benefits for older adults with various health conditions, such as mild cognitive impairment, brain injury, and severe mental illness (Bartels and Pratt, 2009).

To the best of our knowledge, only one RCT study has been conducted to date in middle-aged or older out-patients with schizophrenia (mean age, 46.9 and 48.5 for CR and Control, respectively), and it reported null effects in the neuropsychological assessments of the outpatients compared to active control groups (Dickinson et al., 2010). Since Spaulding et al. (1999a)'s seminal work on CR for inpatients with schizophrenia during intensive PR, numerous RCT trials have demonstrated its clinical effectiveness and functional importance for recovery of relatively younger inpatients with schizophrenia (Medalia et al., 1998, 2000, 2001; Sartory et al., 2005; Silverstein et al., 2005, 2009; Ueland and Rund, 2005; Vauth et al., 2005; Wykes et al., 2007). Despite the growing number of chronic and older inpatients with schizophrenia, only one RCT has been conducted on CR in relatively older in-patients (mean age, 43.46), and its findings suggested that CR results in additional benefits within the context of vocational rehabilitation (Lindenmayer et al., 2008). Previous meta-analyses (McGurk et al., 2007b; Wykes et al., 2011) have emphasized that investigations of whether CR has additive benefits in older patients with schizophrenia within the context of PR are of great interest (Bartels and Pratt, 2009; Sharma et al., 2016).

To examine the effects of CR and PR together and PR only on neurocognitive functioning and psychiatric symptoms in this study, we included a treatment-as-usual (TAU) group (without PR) as another control group. Importantly, to the best of our knowledge, no RCTs have been conducted on non-western inpatients within the context of comprehensive PR, even though inpatient clinics, in conjunction with community-based PR, are important settings for PR.

Thus, in the current study, we employed a RCT design (CR + PR vs. PR only) to evaluate the efficacy of a CR program for middle-aged or older inpatients with chronic schizophrenia within a PR context compared to TAU. We hypothesized that CR + PR treatment would result in the greatest improvements, followed by PR only and TAU, in untrained neurocognitive domains and psychiatric symptoms in middle-aged or older inpatients with chronic schizophrenia.

## Methods

### Clinical trial design

**Figure 1:**
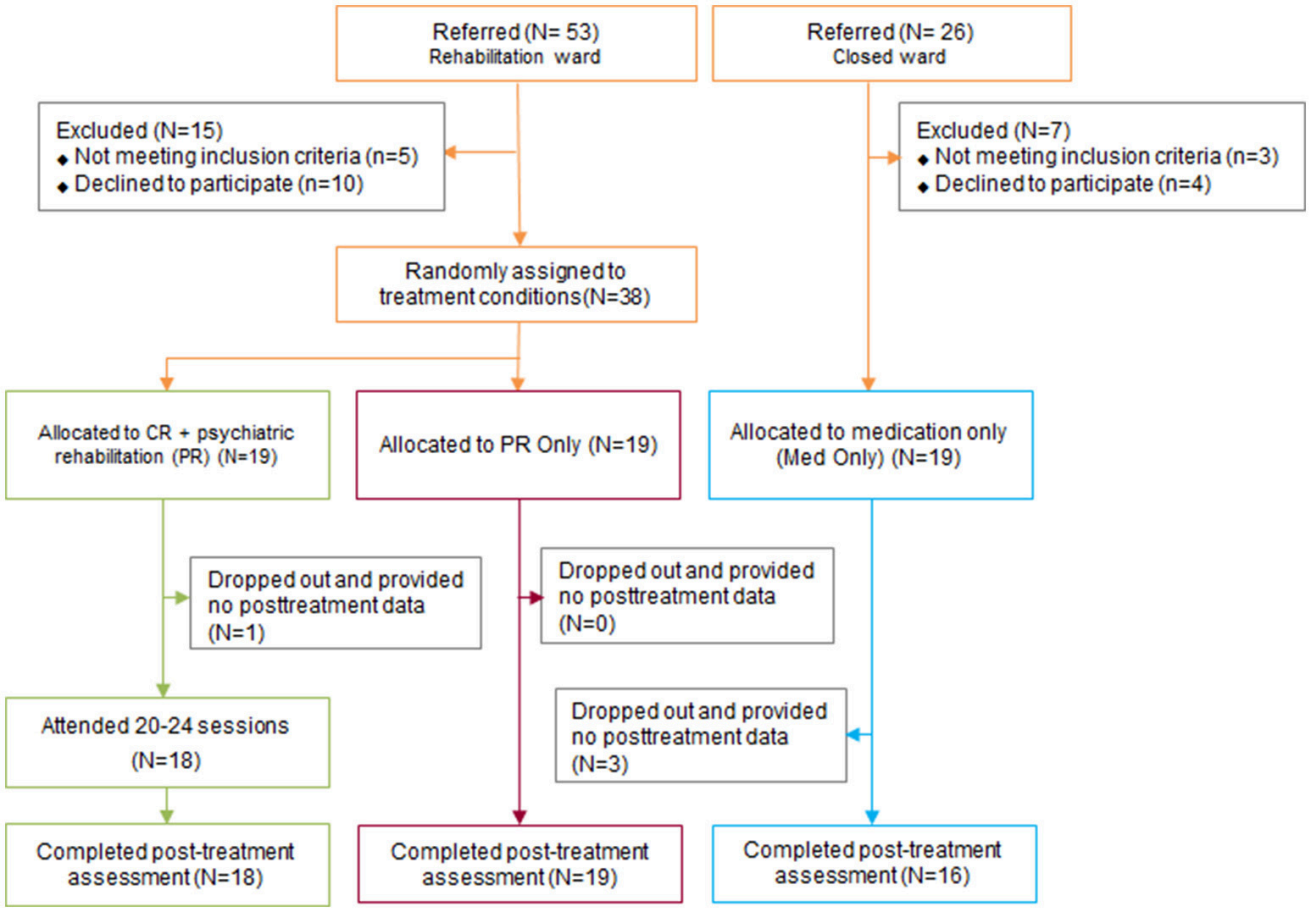
Consolidated standards of reporting trials diagram of the selection of the participants in the study. CR, Cognitive remediation; PR, Psychiatric rehabilitation; TAU, Treatment As Usual.

Of the 79 inpatients with schizophrenia who were referred to this clinical trial (53 from the PR unit and 26 from the TAU unit), eight did not meet the inclusion criteria and 14 declined to participate. A final total of 38 inpatients with schizophrenia from the PR unit who met the inclusion criteria of the study and who gave consent were randomly assigned to the CR + PR or PR-only groups (Figure 1). Nineteen inpatients with schizophrenia from the TAU unit were allocated to the TAU group (Figure 1). The participants from the PR unit who met the study criteria and consented to the current trial were informed that they were assigned to CR in addition to the usual PR programs or the usual PR program only. The TAU group was informed that they would be assessed two different times. All participants were interviewed and assessed before starting the trial and immediately after the trial. Positive and Negative Syndrome Scale (PANSS) and neurocognitive assessments were conducted by master-level pretrained research assistants. The administration and scoring procedures were supervised by licensed clinical psychologists (K. H. Choi, W. H. Lee, and S. Choi). The current study has been registered with Clinical Research Information Service (CRIS) registry, number KCT0002609.

### Participants

The diagnoses of the 57 inpatients with schizophrenia (32 males and 25 females) were confirmed by the Structured Clinical Interview for DSM-IV Axis I disorders (American Psychiatric Association, 1994). Prior to the very recent reform in “Acts on the Improvement of Mental Health and the Support for Welfare Services for Mental Patients” in Korea, the average percentage of involuntary hospitalization to mental health units in Korea has been very high from 67.9 to 93.3% (Ministry of Health Welfare, 2016). All participants in this study were involuntarily admitted to the long stay close unit of the hospital. The mean age of the patients was 50.07, with a range of 36–59, and their number of years of education was 10.93. All patients had been on stable medication regimens for the previous 30 days, with no groups differed for psychotropic medication dose equivalents (Inada and Inagaki, 2015) and maintained their medication dosages during the current 3-month long outcome trial, except for PRN medications. Participants were excluded if they had any of the following: substance use, serious traumatic brain injury or other neurological disorder, or acute psychiatric symptoms. Four patients were dropped due to discharge. All participants in this research voluntarily participated and provided written informed consent.

### Measures

#### Psychiatric symptoms

Psychiatric symptoms were measured by using the PANSS (Kay et al., 1987), which includes five subscales: negative, excitement, cognitive, positive, and depressive (Bell et al., 1994). The internal consistency coefficients of the original version of each subscale were the following: negative (α = 0.86), excitement (α = 0.76), cognitive (α = 0.79), positive (α = 0.80), and depressive (α = 0.69). In the current study, the internal consistency coefficients were of each subscale were the following: negative (α = 0.92), excitement (α = 0.45), cognitive (α = 0.43), positive (α = 0.74), and depressive (α = 0.67). In the current study, the inter-rater reliability coefficient was 0.96.

#### Motivation

Participants' overall motivation was measured by the BIS/BAS scale (Carver and White, 1994; Kim and Kim, 2001).

#### Premorbid IQ

Premorbid IQ was estimated by using the Information subtest score from the Wechsler Adult Intelligence Scale-Fourth edition (WAIS-IV) (Wechsler, 2008) and demographic variables, including years of education, age, gender, and ethnicity (Kim et al., 2015).

#### Neurocognition

Attention/processing speed was assessed by using the Trail Making Test A, which requires the examinee to draw a line between numbers in order within 360 s (Arbuthnott and Frank, 2000). Processing speed was also assessed by using the symbol-coding test, which is a subtest of the WAIS-IV (Wechsler, 2008) and which requires the conversion of numbers to matched symbols in a limited amount of time. Patients with schizophrenia exhibit decreased processing speed and working memory (Kreiner and Ryan, 2001).

Working memory was assessed by using the letter-number sequencing test, which is a subtest of the WAIS-IV (Wechsler, 2008) and which requires the manipulation or visualization of orally presented words and numbers and the recitation of the numbers first in numerical order and then letters in alphabetical order. Verbal logical memory (LM) was assessed by using the LM (I and II) tests, which are subtests of the Wechsler Memory Scale-Fourth Edition (WMS-IV) (Wechsler, 2009). The test is comprised of immediate recall, delayed recall, and recognition tasks for two stories. The stories are read with clear pronunciation by the examiner. The LM tests measure the examinees' verbal memory capacity, which is the ability to recall organized and meaningful linguistic information components.

Executive functioning was assessed by using the Trail Making Test B (Trails B), Wisconsin Card Sorting Test (WCST) 64, and Verbal Fluency Test. The Trails B requires the examinee to draw a line between numbers and letters that are scattered on paper following the given rules within 360 s (Arbuthnott and Frank, 2000). The WCST evaluates executive function, which is the essential cognitive function that enables goal setting, planning, and goal-directed behaviors. The WCST 64 consists of four stimulus cards and 64 response cards that have three different dimensions (i.e., form, color, and number), with each having four components. The examinee is required to place each response card under a stimulus card that they consider a match. After matching the cards, (s)he gets feedback on whether they were right or wrong. Because they are not given any instructions on the pairing rules, the examinees have to determine the rules from the feedback. The verbal fluency test requires the examinees to find as many words as possible that begin with a given consonant in a limited amount of time (60 s) (Lee et al., 2000).

### CR

**Figure 2:**
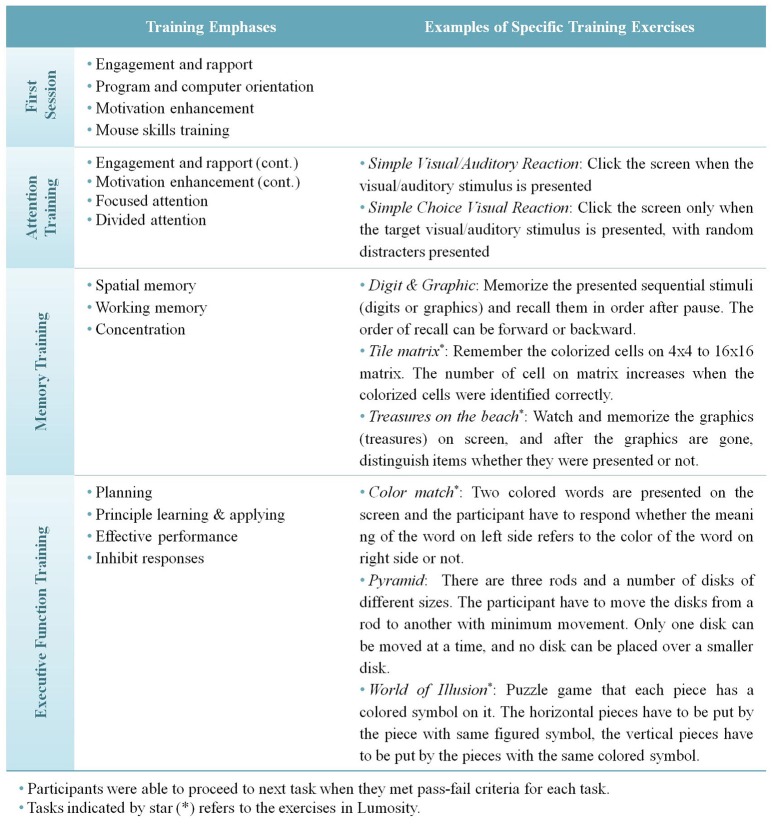
Targeted domains and detailed training tasks in CR.

The CR treatment consisted of 24 sessions that occurred twice a week for 1 h/session for over 3 months. The PSSCogRehab software program (version 12.0; Psychological Software Services, Inc., Indianapolis, IN, USA) was translated in Korean by K. H. Choi's research group and then used for CR training (Bracy, 2012). The CR training was formulated to include practicing attention, memory, and executive functioning (Figure 2). The starting level and initial training schedules were determined for each individual by a therapist according to their pretreatment assessment. The training schedules (e.g., targeted neurocognitive domains, difficulty levels, etc.) were individualized and updated based on the participants' preferences and levels of performance. CR games from Lumosity.com were also employed to supplement the CR training for spatial memory (e.g., tile matrix and treasures on the beach) and executive functioning (e.g., color match and world of illusion). Because most participants in the current study were not familiar with using computers, the therapists introduced the basic skills (e.g., using a mouse) of computer usage at the initial session and then gradually moved to the CR tasks. The CR sessions consisted of computer-based CR training (50 min) and a bridging group session (10 min) (Medalia et al., 2009). To increase motivation, the participants were reminded at the beginning of each session of their goals for joining the CR program, and they were given the opportunity to link their personal goals with the CR training. In addition, the participants were able to choose tasks that they wanted to practice in each session with the clinician's assistance and monitor their progress. After about 50 min of computer-based CR training, the group members gathered for a bridging group (Medalia et al., 2009), in which they discussed the connections between their goals and the cognitive training and exchanged strategies. The therapists used motivational interviewing techniques to enhance the participants' intrinsic motivation toward the CR (Fiszdon et al., 2016; Lee et al., 2017). All CR sessions were reviewed by K. H. Choi in a weekly supervision meeting or *in vivo* training sessions to confirm the consistency of the CR protocol.

### PR

The participants who were randomized to the CR + PR and PR-only groups received comprehensive inpatient PR, including optimal pharmacotherapy, vocational rehabilitation, social skills training, daily living skills training, illness management, independent living skills training, and patient empowerment program. The PR did not include specific training on neurocognitive functioning.

### TAU

The participants who were recruited from the TAU unit received optimal pharmacotherapy, psychoeducation, socialization and recreational programs. The TAU group did not receive specific training on neurocognitive functioning or PR-specific components, such as vocational rehabilitation or skills training.

### Data analysis

A series of repeated-measures analyses of variance [within-subjects factor, time; between-subjects factor, group (CR + PR, PR only, or TAU)] were conducted to examine the effects of CR on the neurocognitive measures, psychiatric symptoms, and clinic unit behaviors. The effect sizes were calculated with the Time-by-Group interaction. *Post-hoc* analyses were conducted to compare the group means at each time point. To estimate the clinical significance, the reliable change index (RCI) was calculated and compared among the groups (Wise, 2004). The RCI was calculated as $\frac{X_{1}-X_{2}}{SE}$ (X_1_ = pretest score; X_2_ = post-test score; SE = $s_{1}\sqrt{1-r_{\text{xx}}}$; s_1_ = the standard deviation of the control group, normal population, or pretreatment group; and r_xx_ = the test-retest reliability). RCI scores that were equal or >1.96 are considered reliable changes (Wise, 2004).

### Power analysis

Using G^*^Power (version 3.1.9.2, Heinrich Heine University, Düsseldorf, Germany) (i.e., repeated measures analyses of variance, measures at two-time points for three groups, *p* < 0.05, correlations = 0.5–0.8, and Cohen's *d* = 0.58 in the context of PR) (Faul et al., 2009), moderate effects were found in 45 participants. Considering the potential for missingness, we assigned 19 participants to each group.

### Ethical standards

The study was approved by both Yongin Mental Hospital Institutional Review Board and Korea University Institutional Review Board. The authors assert that all procedures contributing to this work comply with the ethical standards of the relevant national and institutional committees on human experimentation and with the Helsinki Declaration of 1975, as revised in 2008.

## Results

As shown in Table 1, 38 participants were randomized to either the CR + PR or PR-only groups, and an additional 19 participants from the TAU unit were allocated to the TAU group. No group differences were found in key demographical variables (e.g., age, education, and gender ratio), premorbid IQ, psychiatric characteristics (e.g., age of onset, duration of illness, number of hospitalizations, BIS/BAS scores and PANSS scores), or baseline neurocognitive functioning (Table 1). The drop-out rates were low for the CR + PR (5.3%) and PR (0.0%) participants, while it was 15.8% for the TAU participants (Figure 1).

**Table 1:** Demographic information.

	**CR + PR**	**PR Only**	**TAU**	***F*-value**	**Significance**
	***n* = 19**	***n* = 19**	***n* = 19**	**Or χ*^2^***	**(*p*-value)**
	**M (SD)**	**M (SD)**	**M (SD)**		
Age (years)	49.58 (6.19)	49.74 (6.07)	50.89 (6.00)	0.26	0.77
Education (years)	11.11 (3.14)	11.26 (3.72)	10.42 (2.69)	0.37	0.69
Gender, male (percentage)	63.16	52.63	52.63	0.57	0.75
Premorbid IQ estimate	94.37 (9.05)	97.55 (10.57)	93.92 (9.15)	0.80	0.45
Age of Onset	27.81 (7.31)	25.87 (6.84)	23.45 (6.86)	1.80	0.17
Duration of Illness	22.19 (10.66)	23.87 (7.94)	27.45 (5.89)	1.93	0.16
Number of Hospitalizations	5.05 (4.01)	4.67 (3.31)	6.37 (7.22)	0.59	0.58
**PSYCHIATRIC SYMPTOMS**					
**PANSS**
Cognition	23.11 (6.26)	21.32 (3.96)	23.53 (5.58)	0.91	0.41
Depression/Anxiety	11.58 (5.23)	10.11 (4.19)	8.89 (2.98)	1.91	0.16
Excitement/Hostility	5.74 (1.88)	5.84 (1.68)	6.74 (2.21)	1.53	0.22
Negative	16.58 (5.43)	15.37 (5.96)	17.37 (7.22)	0.49	0.61
Positive	13.32 (6.28)	12.16 (5.04)	12.21 (4.39)	0.29	0.75
**COGNITIVE FUNCTIONS (T SCORES)**					
**Processing Speed**					
Coding	37.72 (11.11)	39.12 (13.19)	34.21 (10.71)	0.89	0.42
**Attention**					
TMT-A	52.67 (10.34)	55.02 (7.52)	48.67 (21.27)	0.96	0.39
**Verbal Working Memory**					
LNS	37.89 (14.19)	36.67 (9.36)	32.63 (12.75)	0.96	0.39
**Verbal Memory**					
LM I	32.98 (9.42)	36.67 (11.39)	31.93 (6.88)	1.33	0.27
LM II	31.4 (9.05)	33.33 (11.86)	28.07 (5.59)	1.59	0.21
**Executive Function**					
TMT-B	37.12 (14.1)	34.22 (12.96)	28.73 (10.19)	2.20	0.12
VFT	10.63 (3.53)	10.21 (3.05)	9.32 (2.87)	0.86	0.43
WCST TE	36.84 (11.3)	35.58 (12.33)	34.95 (10.11)	0.14	0.87
WCST %PE	40.13 (20.31)	34.52 (22.44)	35.78 (23.81)	0.33	0.72
WCST %CL	25.76 (22.78)	29.86 (22.23)	29.79 (18.19)	0.23	0.79
**CPZ Equivalents**	1243.79 (2772.51)	2517.99 (6048.33)	510.43 (487.78)	1.32	0.28
**BIS/BAS**					
Reward Responsiveness	14.16(2.77)	14.47(2.53)	13.74(2.79)	0.36	0.70
Drive	11.47(2.29)	10.89(2.23)	10.68(2.69)	0.55	0.58
Fun Seeking	9.89(2.38)	10.89(1.78)	9.26(2.49)	2.56	0.09
Behavioral Inhibition	18.63(2.85)	19.42(2.06)	19.21(3.19)	0.42	0.66

*CR, Cognitive remediation; PR, Psychiatric rehabilitation; TAU, Treatment as usual; M, mean; SD, standard deviation; PANSS, Positive and Negative Syndrome Scale; TMT-A, Trail Making Test-A; LNS, Letter-Number Sequencing; LM I, Logical Memory I; LM II, Logical Memory II; TMT-B, Trail Making Test-B; VFT, Verbal fluency test; WCST TE, Wisconsin Card Sorting Test Total Errors; WCST %PE, Wisconsin Card Sorting Test Perseveration errors; WCST %CL, Wisconsin Card Sorting Test Conceptual level responses; CPZ, chlorpromazine*.

### Effects of CR on neurocognition

The data for all neurocognitive variables are presented in Table 2. The time (i.e., pre- and post-treatment) × group (i.e., CR + PR, PR only, and TAU) interactions were significant for both immediate recall (*p* < 0.001, $\eta_{p}^{2}$ = 0.21) and delayed recall (*p* = 0.03, $\eta_{p}^{2}$ = 0.13), with medium to large effect sizes. The total errors, perseverative errors, and conceptual level (CL) responses (%) were analyzed to observe abstract thinking, learning strategies, and cognitive flexibility. The time × group interactions were significant for WCST total errors (*p* = 0.02, $\eta_{p}^{2}$ = 0.15) and CL responses (*p* = 0.004, $\eta_{p}^{2}$ = 0.20), with medium to large effect sizes (Table 2). No group differences were found for processing speed (WAIS-IV Coding), attention (TMT-A), verbal working memory (WAIS-IV Letter-Number Sequencing), or cognitive flexibility (WCST perseveration errors).

**Table 2:** Effects of cognitive remediation on neurocognition.

**Outcome measures**		**CR** + **PR**		**PR Only**		**TAU**		**Group × Time**	**ES**
		***n*** = **18**		***n*** = **19**		***n*** = **16**		***p*-value**	***($\eta_{p}^{2}$)***
		**Pre**	**Post**	**Pre**	**Post**	**Pre**	**Post**		
		**M (SD)**	**M (SD)**	**M (SD)**	**M (SD)**	**M (SD)**	**M (SD)**		
**PROCESSING SPEED**									
	Coding	38.70 (10.55)	39.44 (11.73)	39.12 (13.19)	41.58 (14.20)	32.92 (10.81)	31.46 (8.52)	0.13	0.08
**ATTENTION**									
	TMT-A	52.81 (10.62)	55.58 (12.03)	55.02 (7.52)	57.76 (6.51)	47.98 (22.79)	46.00 (22.94)	0.52	0.03
**VERBAL WORKING MEMORY**									
	LNS	38.89 (13.91)	41.30 (10.91)	36.67 (9.36)	39.12 (10.23)	31.25 (13.44)	29.38 (10.63)	0.16	0.07
**VERBAL MEMORY**									
	LM I	33.15 (9.67)	41.30 (13.19)	36.67 (11.39)	37.02 (11.49)	32.08 (7.39)	29.79 (9.23)	<0.01	0.21
	LM II	31.85 (9.09)	40.37 (13.52)	33.33 (11.86)	35.61 (13.43)	27.71 (5.67)	28.75 (7.59)	0.03	0.13
**EXECUTIVE FUNCTION**									
	TMT-B	37.28 (14.49)	37.16 (14.36)	34.22 (12.96)	40.08 (9.31)	29.53 (10.33)	33.31 (11.15)	0.14	0.07
	VFT	10.83 (3.52)	10.83 (3.4)	10.21 (3.05)	10.79 (2.49)	9.00 (2.97)	9.69 (3.16)	0.59	0.02
	WCST TE	36.17 (11.23)	30.67 (12.33)	35.58 (12.33)	38.53 (8.74)	36.81 (9.57)	39.63 (9.79)	0.02	0.15
	WCST %PE	40.48 (20.84)	34.11 (21.99)	34.52 (22.44)	39.14 (20.94)	39.07 (24.56)	44.14 (24.07)	0.17	0.07
	WCST %CL	27.19 (22.54)	36.46 (25.62)	29.86 (22.23)	19.98 (16.15)	26.98 (17.69)	20.21 (17.38)	<0.01	0.20

*CR, Cognitive remediation; PR, Psychiatric rehabilitation; TAU, Treatment as usual; ES, Effect size; $\eta_{\text{p}}^{2}$, Partial eta square; M, mean; SD, standard deviation; TMT-A, Trail Making Test-A; LNS, Letter-Number Sequencing; LM I, Logical Memory I; LM II, Logical Memory II; TMT-B, Trail Making Test-B; VFT, Verbal fluency test; WCST TE, Wisconsin Card Sorting Test Total Errors; WCST %PE, Wisconsin Card Sorting Test Perseveration errors; WCST %CL, Wisconsin Card Sorting Test Conceptual level responses*.

Least Significant Difference *post-hoc* tests were conducted on the variables with significant time × group interactions (i.e., LM I & II, WCST total errors, and WCST %CL) to investigate whether the groups differed at post-treatment (Supplemental Tables 1, 2). The CR + PR group showed greater post-treatment performance on both LM I and II compared with the performance of the TAU group (LM I: mean difference = 11.50, *p* = 0.005; LM II: mean difference = 11.62, *p* = 0.007), while the CR + PR and PR-only groups did not differ in both LM I and II. The PR-only group showed greater performance at a trend level of significance compared with the TAU group (LM I: mean difference = 7.23, *p* = 0.070; LM II: mean difference = 6.86, *p* = 0.098).

**Figure 3:**
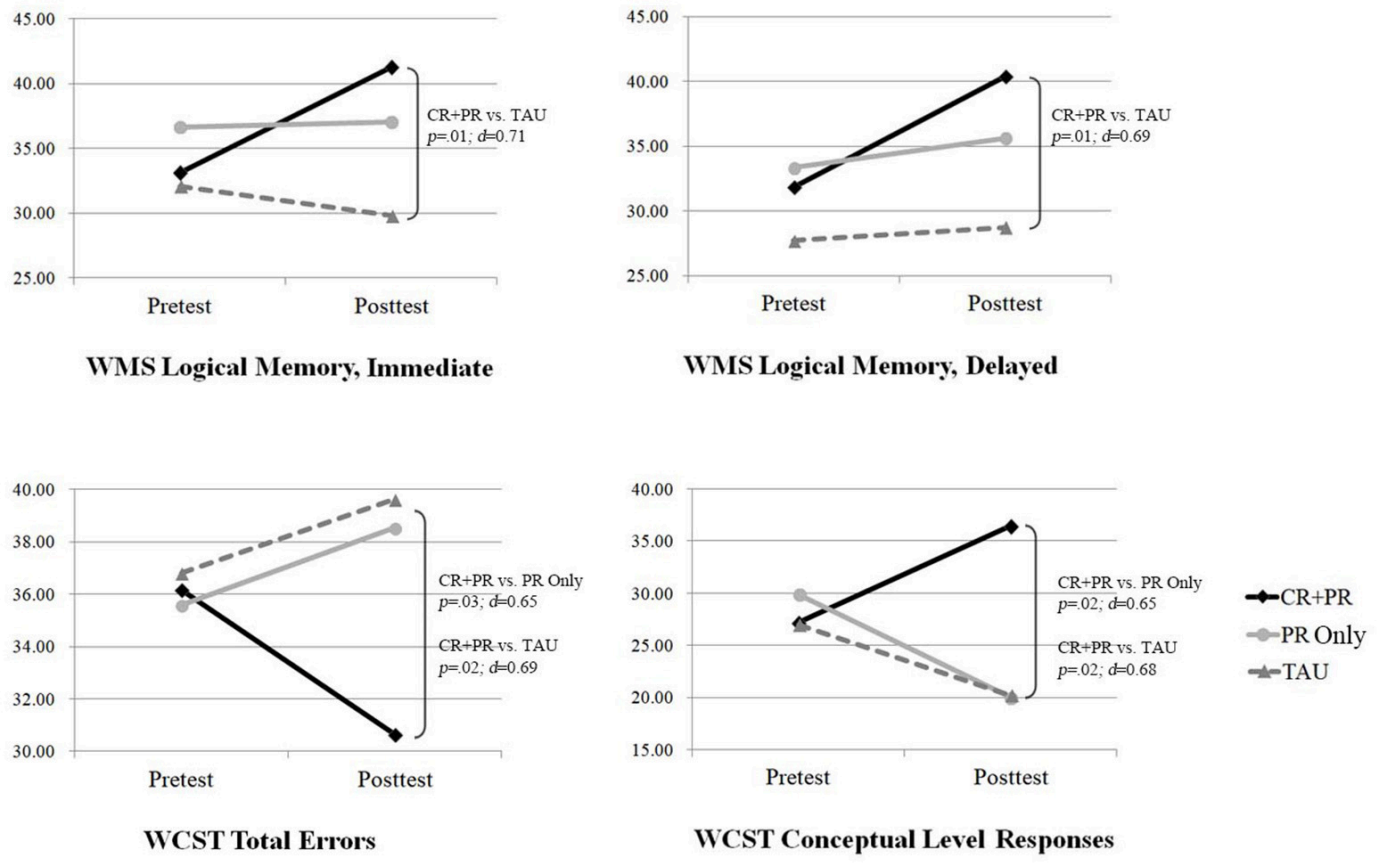
Effects of CR on logical memory and executive functioning.

In addition, the CR + PR group showed greater post-treatment performance on both WCST total errors and WCST %CL compared with the PR-only group (WCST total errors: mean difference = 12.28, *p* = 0.026; WCST %CL: mean difference = 16.47, *p* = 0.017) and TAU group (WCST total errors: mean difference = 14.00, *p* = 0.015; WCST %CL: mean difference = 16.24, *p* = 0.023). However, no group differences were found on WCST total errors and WCST %CL between the PR-only and TAU groups (Figure 3).

### Effects of CR in psychiatric symptoms

No significant group differences were observed for psychiatric symptoms (PANSS scores) (Table 3).

**Table 3:** Effects of cognitive remediation on psychiatric symptoms.

**Psychiatric symptoms**	**CR** + **PR**		**PR Only**		**TAU**		**Group × Time**	**ES**
	***n*** = **18**		***n*** = **19**		***n*** = **16**		***p*-value**	**(*$\eta_{p}^{2}$*)**
	**Pre**	**Post**	**Pre**	**Post**	**Pre**	**Post**		
	**M (SD)**	**M (SD)**	**M (SD)**	**M (SD)**	**M (SD)**	**M (SD)**		
**PANSS**									
Cognition	23.11 (6.26)	22.79 (8.03)	21.32 (3.96)	22.42 (4.57)	23.53 (5.58)	21.58 (11.98)	0.56	0.02
Depression/Anxiety	11.58 (5.23)	9.42 (4.82)	10.11 (4.19)	9.84 (3.2)	8.89 (2.98)	8.26 (4.93)	0.44	0.03
Excitement/Hostility	5.74 (1.88)	5.95 (2.44)	5.84 (1.68)	6.05 (1.9)	6.74 (2.21)	6.95 (4.36)	1.00	0.00
Negative	16.58 (5.43)	15.58 (7.43)	15.37 (5.96)	19.05 (6.07)	17.37 (7.22)	17.68 (10.07)	0.12	0.08
Positive	13.32 (6.28)	12.26 (6.13)	12.16 (5.04)	12.53 (5.49)	12.21 (4.39)	11.05 (6.32)	0.58	0.02

*CR, Cognitive remediation; PR, Psychiatric rehabilitation; TAU, Treatment as usual; ES, Effect size; $\eta_{\text{p}}^{2}$, Partial eta square; M, mean; SD, standard deviation; PANSS, Positive and Negative Syndrome Scale*.

### RCI

**Figure 4:**
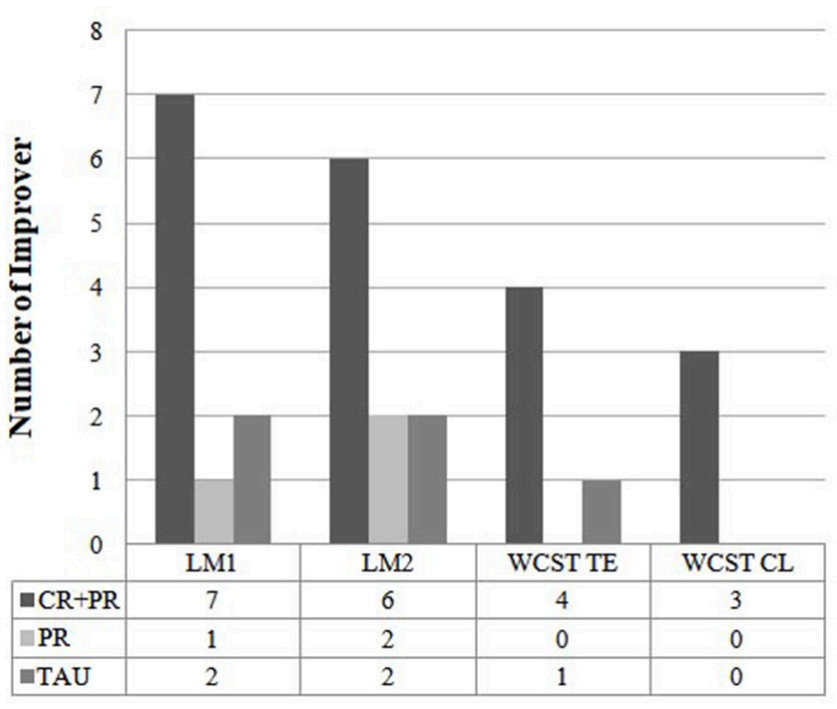
Comparisons of the number of participants with meaningful improvement.

The RCIs were calculated for LM I and II, WCST total errors, and WCST %CL, which showed significant time × group interactions. As recommended by Wise (Wise, 2004), RCI scores ≥1.96 were considered reliable changes. As shown in Figure 4, more participants in the CR + PR group showed clinically significant improvements for the LM I (CR + PR: 7 participants; PR only: 1 participant; TAU: 2 participants) and LM II (CR + PR: 6 participants; PR only: 2 participants; TAU: 2 participants) (LM I: χ^2^ = 8.35, *p* = 0.02; LM II: χ^2^ = 6.92, *p* = 0.03). Differences with trend levels of significance were found for WCST total errors (CR + PR: 4 participants; PR only: 0 participants; TAU: 1 participant) and WCST %CL (CR + PR: 3 participants; PR only: 0 participants; TAU: 0 participants) (WCST total errors: χ^2^ = 5.18, *p* = 0.08; WCST %CL: χ^2^ = 5.67, *p* = 0.06).

## Discussion

The current study aimed to investigate whether CR treatment within the context of PR treatment (CR + PR) would produce meaningful improvements in neurocognition and psychiatric symptoms in middle-aged or older inpatients with chronic schizophrenia compared to either the PR-only or TAU group. For the CR, we employed a highly personalized and computerized CR protocol with motivational enhancements through a bridging group and motivational interviewing. To the best of our knowledge, the current study is the first RCT of middle-aged or older inpatients with chronic schizophrenia compared with PR-only and TAU groups, especially conducted in a non-western country.

The results of the current study partially supported our primary hypothesis. Specifically, compared with the PR-only and TAU groups, the CR + PR group showed greater improvement in executive functioning (e.g., abstract thinking and learning strategies). In addition, the CR + PR group showed greater improvements in immediate and delayed LM compared with the TAU group but not the PR-only group. The PR-only group also showed greater improvements in immediate and delayed LM compared with the TAU group. The PR group improved more in executive functioning than the TAU did, but the difference was not reach significant. Importantly, the RCIs indicated that more participants in the CR + PR group had clinically meaningful improvements in LM and executive functioning compared with participants in the PR-only and TAU groups. However, since TAU group was not randomly assigned, the differences between “CR+PR vs. TAU” or “PR-only vs. TAU” should not be interpreted as causation.

These findings suggest that CR has additional treatment benefits for executive-level operation when it is delivered within a larger context of PR compared with PR only. Inpatient PR has nonspecific treatment effects on neuropsychological functioning, even without explicit CR training (Spaulding et al., 1999b). In addition to the nonspecific treatment effects of PR, CR effectively stimulates neurocognition to synergistically accelerate the benefits of PR.

No group differences were found in other neurocognitive domains or psychiatric symptoms. The pattern of treatment gains found in the current study was somewhat similar to the results of a previous controlled outcome trial that targeted younger patients in a comprehensive and intensive inpatient PR setting (Spaulding et al., 1999a) and that reported significant treatment gains in Card Sort random errors but not perseverative errors, Trails B, and PANSS total and subscales. The null effects of CR in other neurocognitive domains and psychiatric symptoms could be interpreted in several ways. All participants in the current trial received optimal pharmacotherapy, supportive therapies, and case management, which resulted in nonspecific treatment gains in neurocognition and/or psychiatric symptoms. In addition, the relatively low dose of CR (i.e., 24 sessions, twice per week) and small sample (i.e., 19 per group) in the current study might have resulted in insufficient power for detecting any potential treatment gains.

Comparing the current findings with the results of a previous RCT that was conducted in outpatients with similar ages (Dickinson et al., 2010) shows that, unlike the previous study, the current study found treatment gains in untrained neuropsychological assessments (i.e., executive functioning). Important differences between these two studies need to be acknowledged when interpreting the results. First, the patients examined in the current study were chronic inpatients who participated in the usual inpatient PR-only (without CR) and TAU units. Compared to outpatient clinics, inpatient settings have less cognitive stimulation. Thus, the addition of CR to inpatient PR might result in additional improvements in neuropsychological assessments compared with control conditions. Second, unlike Dickinson et al. (2010), we did not include active computer skills training as a control condition, and this might have resulted in greater differences between the CR + PR and PR-only groups in our study compared with those in the study by Dickinson et al. (2010).

The effects of CR on the neuropsychological assessments in the middle-aged or older inpatients with chronic schizophrenia in the current study were in line with the outcomes of previous CR studies. Bowie et al. have reported that both younger (early-course group) and older outpatients (longer-course group) with schizophrenia improved in working memory (digit sequencing) and executive functioning (Tower of London), but only the improvements in the early-course group in executive functioning were statistically significant and the improvements in working memory trended to significance. These findings suggested that older inpatients with schizophrenia maintain neuronal plasticity. Thus, CR should be considered as an adjunct treatment to usual PR services.

This study had several limitations. The CR training sessions were designed to have a relatively low dose (i.e., 24 sessions, twice per week for about 3 months) due to the durations of inpatient hospitalizations. Even though the low CR dose was similar to those used in previous studies (van der Gaag et al., 2002; Lindenmayer et al., 2008), more intense CR training might have produced better treatment outcomes. We did not have follow-up assessments also because of the durations of inpatient hospitalizations. Thus, the durability of the treatment gains should be explored in an inpatient PR setting in a longer future study. We should also acknowledge that the half of our evaluators were not blinded completely in measuring psychiatric symptoms or neurocognition in this study. To minimize potential evaluators' bias, all evaluators were trained intensively to approximate a great inter-rater reliability. During the administration and scoring process, they were observed and supervised with verbatim process by licensed psychologists (KHC, WHL, SC). Nevertheless, the evaluators' bias cannot be negated. Importantly, even though our sample size was modest for detecting medium effects, the statistical power of this study might have been insufficient for detecting small to moderate treatment gains. Thus, additional studies with more patients and longer CR sessions should be conducted to attempt to replicate the current findings. Finally, the effects of such improvements in cognitive function on daily function or social functioning in the actual ward need further investigation.

Despite the limitations noted above, the results of the current study highlight the importance of delivering CR within a PR context to middle-aged or older inpatients with chronic schizophrenia, especially to improve their executive functioning, which is a critical factor for learning in PR and treatment (Green, 1996; Spaulding et al., 1999a; Bowie and Harvey, 2006).

## Author contributions

K-HC, S-HL, and T-YH designed the study. K-HC wrote the first draft of the manuscript and supervised cognitive remediation sessions, S-MK and JK administered cognitive remediation. S-MK, JK, and KP undertook the statistical analysis. S-CP supervised participants' recruitment and administration of psychotropic medications during trials. W-HL and SC supervised neurocognitive and clinical assessments. All the authors commented on the manuscript. All the authors contributed to and have approved the final manuscript.

### Conflict of interest statement

The authors declare that the research was conducted in the absence of any commercial or financial relationships that could be construed as a potential conflict of interest.

## Abbreviations

CL: Conceptual level
CR: Cognitive remediation
LM: Logical memory
PANSS: Positive and negative syndrome scale
PR: Psychiatric rehabilitation
RCI: Reliable change index
RCTs: Randomized controlled trials
TAU: Treatment as usual
WAIS-IV: Wechsler Adult Intelligence Scale-Fourth edition
WCST: Wisconsin card sorting test
WMS-IV: Wechsler Memory Scale-Fourth Edition.
