# Supplementary material for: Cognitive Remediation in Middle-Aged or Older Inpatients with Chronic Schizophrenia: A Randomized Controlled Trial in Korea
Source: Front Psychol. 2018 Feb 6;8:2364. doi: 10.3389/fpsyg.2017.02364 (PMC5807907; doi:10.3389/fpsyg.2017.02364)
Supplement: Supplementary file 1 [file Table1.docx]

Supplement Table 1. *Post-hoc test results at post-treatment*

|  |  | Sum of Squares | df | Mean Square | F | *p* |
| --- | --- | --- | --- | --- | --- | --- |
| LM1 | Between Groups | 1136.84 | 2 | 568.42 | 4.30 | 0.02 |
|  | Within Groups | 6611.16 | 50 | 132.22 |  |  |
|  | Total | 7748.01 | 52 |  |  |  |
|  |  |  |  |  |  |  |
| LM2 | Between Groups | 1149.99 | 2 | 574.99 | 3.98 | 0.02 |
|  | Within Groups | 7218.14 | 50 | 144.36 |  |  |
|  | Total | 8368.13 | 52 |  |  |  |
|  |  |  |  |  |  |  |
| WCST TE | Between Groups | 841.63 | 2 | 420.81 | 3.90 | 0.03 |
|  | Within Groups | 5398.49 | 50 | 107.97 |  |  |
|  | Total | 6240.11 | 52 |  |  |  |
|  |  |  |  |  |  |  |
| WCST % CL | Between Groups | 3185.48 | 2 | 1592.74 | 3.91 | 0.03 |
|  | Within Groups | 20385.98 | 50 | 407.72 |  |  |
|  | Total | 23571.45 | 52 |  |  |  |

Supplement Table 2. *LSD comparisons*

|  |  |  | |  |  | 95% Confidence  Interval | |  |  |
| --- | --- | --- | --- | --- | --- | --- | --- | --- | --- |
|  | (I)  Group 1 | | (J)  Group 2 | Mean  Diff (I-J) | Std.  Error | Lower  Bound | Upper  Bound | *p* | |
| LM1 | CR+PR | | PR Only | 4.28 | 3.78 | -3.32 | 11.88 | 0.26 | |
|  |  | | TAU | 11.50 | 3.95 | 3.57 | 19.44 | 0.01 | |
|  |  | |  |  |  |  |  |  | |
|  | PR Only | | CR+PR | -4.28 | 3.78 | -11.88 | 3.32 | 0.26 | |
|  |  | | TAU | 7.23 | 3.90 | -0.61 | 15.06 | 0.07 | |
|  |  | |  |  |  |  |  |  | |
|  | TAU | | CR+PR | -11.50 | 3.95 | -19.44 | -3.57 | 0.01 | |
|  |  | | PR Only | -7.23 | 3.90 | -15.06 | 0.61 | 0.07 | |
|  |  | |  |  |  |  |  |  | |
| LM2 | CR+PR | | PR Only | 4.76 | 3.95 | -3.18 | 12.69 | 0.23 | |
|  |  | | TAU | 11.62 | 4.13 | 3.33 | 19.91 | 0.01 | |
|  |  | |  |  |  |  |  |  | |
|  | PR Only | | CR+PR | -4.76 | 3.95 | -12.69 | 3.18 | 0.23 | |
|  |  | | TAU | 6.86 | 4.08 | -1.32 | 15.05 | 0.10 | |
|  |  | |  |  |  |  |  |  | |
|  | TAU | | CR+PR | -11.62 | 4.13 | -19.91 | -3.33 | 0.01 | |
|  |  | | PR Only | -6.86 | 4.08 | -15.05 | 1.32 | 0.10 | |
|  |  | |  |  |  |  |  |  | |
| WCST % TE | CR+PR | | PR Only | -7.86 | 3.42 | -14.72 | -0.99 | 0.03 | |
|  |  | | TAU | -8.96 | 3.57 | -16.13 | -1.79 | 0.02 | |
|  |  | |  |  |  |  |  |  | |
|  | PR Only | | CR+PR | 7.86 | 3.42 | 0.99 | 14.72 | 0.03 | |
|  |  | | TAU | -1.10 | 3.53 | -8.18 | 5.98 | 0.76 | |
|  |  | |  |  |  |  |  |  | |
|  | TAU | | CR+PR | 8.96 | 3.57 | 1.79 | 16.13 | 0.02 | |
|  |  | | PR Only | 1.10 | 3.53 | -5.98 | 8.18 | 0.76 | |
|  |  | |  |  |  |  |  |  | |
| WCST % CL | CR+PR | | PR Only | 16.47 | 6.64 | 3.13 | 29.81 | 0.02 | |
|  |  | | TAU | 16.24 | 6.94 | 2.31 | 30.18 | 0.02 | |
|  |  | |  |  |  |  |  |  | |
|  | PR Only | | CR+PR | -16.47 | 6.64 | -29.81 | -3.13 | 0.02 | |
|  |  | | TAU | -0.23 | 6.85 | -13.99 | 13.53 | 0.97 | |
|  |  | |  |  |  |  |  |  | |
|  | TAU | | CR+PR | -16.24 | 6.94 | -30.18 | -2.31 | 0.02 | |
|  |  | | PR Only | 0.23 | 6.85 | -13.53 | 13.99 | 0.97 | |
